# Hospital and Institutional News

**Published:** 1915-09-04

**Authors:** 


### HOSPITAL AND INSTITUTIONAL NEWS.

### THE NEED OF MORE WOMEN DOCTORS.

Mr. F. D. Ackland, speaking at Harrogate on August 30 in support of the appeal for funds for the extension of the London School of Medicine for Women, emphasised the need for more medical women. Because of the lack of doctors there were, he said, hospitals in London which might have to be closed, and they were now beginning to realise the value of women doctors. It is a pity this was not learnt long ago. We deal with the subject of women in medicine in a special article in this issue.

### POOR-LAW MEDICAL OFFICERS AND THE COST OF DRUGS.

The great increase in the cost of many drugs in common use is affecting severely those Poor-Law medical officers who are required by their contracts with the Guardians to supply drugs. At Havant the workhouse medical officer, whose salary is £40 per annum, pointed out to the Guardians that two of the patients in the workhouse were receiving drugs which cost him 2s. a day. At Louth the medical officer has given notice that he must resign unless the Guardians are prepared to make him some allowance to meet the extra cost of his drug bill. Similar applications have been made in other Unions. It is obvious that in all Unions in which the medical officer has to supply drugs some provision must be made to deal with this difficulty. As we have pointed out before, the system under which the medical officer contracts to supply drugs is a vicious one and should be entirely abolished.

### BRISTOL ROYAL INFIRMARY.

Bristol Infirmary, like most of the voluntary hospitals, is suffering from the usual hospital consequences of the war—a diminished staff, shortness of funds, and the pressure produced by the influx of sick and wounded soldiers. The latest report shows that, so far as the civilian public is concerned, the in-patients during the last half-year have fallen off to the extent of 1,000, while, very significantly, the out-patients have increased by some 4,000. The military demands are shown by the admission during the six months of 2,000 soldiers, and when the figures are thus placed in juxtaposition it is evident to what an extent the use of the civil hospitals by the War Office is being practised to the prejudice of the civil population. The arrangement may be an inevitable one, though we strongly doubt it; but, in any event, it is a misfortune for many a poor patient, and it is to use the civil hospitals for a purpose other than the one for which they were intended. The finances at Bristol show an increase in the revenue deficit and an adverse balance on the building account of nearly £23,000.

### SIR WILLIAM THOMAS AND PORTH COTTAGE HOSPITAL.

The munificence of really generous men would seem to be unceasing. Only a few weeks ago the foundation-stone of the new physiological buildings at Cardiff was laid, buildings which, with the completed medical school, will probably cost the donor, Sir William James Thomas, of Ynyshir, a sum not far short of £100,000. It is now reported that it had come to Sir William's knowledge that the Cottage Hospital at Porth was severely taxed in its efforts to meet the immediate requirements of accident cases amongst the miners of the Rhondda Valley, and he has agreed to add a new wing to the existing building, providing accommodation for ten or twelve beds, and to remodel the kitchen arrangements, at an expenditure of about £5,000. This agreement is subject to the condition that the miners themselves should, by a levy, contribute funds sufficient for the staffing and maintenance of the hospital. It is calculated that a subscription of ½d. in the £ would prove sufficient for this purpose, although it is hoped that for the first year the levy may be 1d. in the £, which would enable a reserve fund to be provided. We hope to hear that the miners of the Rhondda Valley, with the good offices of the miners' organisations, have agreed to accept the conditional gift of Sir William Thomas.

### STORING COAL FOR LONDON INFIRMARIES.

Several Metropolitan Boards of Guardians which are desirous of stocking a quantity of coal to meet in a measure their requirements for the coming winter in case there should be a shortage, have been met with the difficulty that they are without a suitable place to keep it in. This was the case with the Wandsworth Board, which was recommended by the St. James's Infirmary Committee to procure 200 tons of steam coal as such a reserve. The committee suggested that it should be stacked on the grass outside the married quarters in an adjoining branch institution; but the chairman, Mr. Percy Rees, thought this would be very unwise, as the coal would be unprotected, and the dust from it would blow into the old folks' rooms. Mr. J. Norman said this was the only decent bit of ground near the building with a tree on it, and consequently it would be a shame to spoil it. Whilst generally acquiescing in the proposal that the reserve stock should be procured, the Guardians felt that another place must be found for the storage. The committee find themselves in a difficulty, owing to a mistaken policy in not having originally secured ample open spaces at their institutions. It is clearly the duty of the Board to follow the precedent set them by that of Camberwell, and to rent forthwith a small site for storing purposes.

### CHOLERA IN GERMANY AND RUSSIA.

A report in the daily Press states that a quarantine notice has been issued from Lloyd's quoting a Foreign Office telegram for the statement that cholera is reported to be prevalent at numerous districts in Germany, also at Riga and Petrograd. This must be read in conjunction with a Reuter telegram from Zurich to the effect that Asiatic cholera is raging in Hungary, and that there is an unusually large percentage of deaths. Up to the time this report appeared, cholera had been remarkably rare in the countries engaged in the war, and, as is well known, in Germany and Russia stringent prophylactic measures had been taken against this devastating disease. As regards Hungary, the Bacteriological Institute of the Budapest Royal Scientific University distributes free serum for the inoculation of those who are exposed to the danger of cholera infection. The inoculation does not confer absolute safety, and it is necessary that those who have been treated in this way should adhere strictly to the hygienic rules for prevention. If, in spite of these precautions, the epidemic has become serious, it will tax medical ingenuity to cope with it. Incidentally it may be recalled that remarkable effects have been obtained by the treatment of cholera with emetine—an alkaloid contained in ipecacuanha root. In a severe epidemic 75 per cent. of recoveries were obtained with emetine administered hypodermically.

### THE YARMOUTH HOSPITAL AND THE GUARDIANS.

The refusal of the Yarmouth Hospital to admit a child sent by the East and West Flegg Board of Guardians was the subject of an acrimonious debate at the last meeting of the Guardians. As is usual in cases of this kind, both parties to the dispute would appear to be to blame. The Guardians failed to comply with the hospital regulations governing admissions, and, when a bed was allotted them, delayed sending the case for several days, during which time the bed had been filled with another case. On the other hand, the case when presented for admission was examined by the house surgeon, who described it as a cretin and unsuitable for hospital treatment. This diagnosis was disputed by the medical officer of the workhouse, who considered the condition of the patient to be the result of neglect and requiring treatment by massage. He stated that the patient had been admitted at once to the Jenny Lind Hospital at Norwich and was improving. The responsibility for refusing a case sent to a hospital by a public body on the advice of its medical officer should not rest with a house surgeon. As a matter of courtesy between two public bodies, a patient sent in this way should not be refused until seen by a member of the visiting staff. But no institution can be administered satisfactorily unless it insists upon its regulations being observed. Boards of Guardians should be the first to realise this, and should require their officers, in their dealings with other public bodies, to adhere punctiliously to any regulations made by such bodies.

### MEDICAL STUDENTS AND THE WAR.

The Admiralty and the War Office have had the most generous, self-denying, whole-hearted co-operation and help from the voluntary hospitals and medical schools throughout the country. It is matter, therefore, for surprise and regret that the War Office should in any form or shape countenance junior medical students taking combatant commissions. Sir Philip Magnus, in his letter to the *Times*, points out that the Under-Secretary of State for War, Mr. Tennant, on June 21 last stated in reply to a question: "It is clear that, if students in their first or second year join the combatant ranks in very large numbers, there will probably be for some years after the war a serious scarcity of doctors. Any expression, therefore, of official opinion which might seem to place on them the obligation of taking up immediate military duty would hardly be in the interests of the Army or of the community as a whole." This answer embodies a clear statement of the position, and we hope that the Secretary of State will cause his reply of August 1 to Professor Halliburton, of King's College, to be withdrawn, with the announcement that on consideration the War Office will not be willing to grant combatant commissions to junior medical students. Professor Halliburton's view that medical students, like munition workers, should not be called upon to go to the Front must be upheld unless the War Office are prepared deliberately to enforce a policy which may prove largely destructive to the efficient maintenance of British hospitals, owing to a shortage in the workers. In view of the approaching session of the medical schools we hope parents and guardians, as well as the younger men who contemplate a medical career, will recognise that their duty lies in pursuing that career and so doing their part to strengthen the forces of the Empire, whilst they leave to others the privilege of taking combatant commissions, failing their engagement as munition workers or in some other field which the Government may decide will better enable them to do their work for the Empire during the continuance of the great war.

### THE "COLLAPSE" OF THE TUBERCULOSIS SERVICE.

Difficulties are being experienced by the Middlesex County Council in carrying out their anti-tuberculosis work owing to the absence of some of their tuberculosis officers, whom it has been found impossible to replace. This kind of experience is, of course, now a common one, and there is every reason to suppose that in the near future much of the tuberculosis work in the country will have to be considerably curtailed, as more and more of these usually young tuberculosis physicians are absorbed into the Army. In Middlesex there are now only three tuberculosis officers remaining, and at a recent meeting when the application for leave of absence for military duty by Dr. Dobson was considered, the County Medical Officer reported failure to obtain a suitable substitute. He at the same time suggested that if the work had to be carried out by the depleted medical staff, it would be necessary to reduce the attendances of the doctors at each dispensary, to cease examining "contacts," and to decrease the number of non-insured patients. Intimation of these changes would have to be sent to Medical Officers of Health, to Guardians, to the Invalid Children's Aid Association, and other bodies. The Public Health Committee decided that the facts should be laid before the Local Government Board, and curiously enough the committee also forwarded a request to the Board asking if the need of Dr. Dobson's services by the War Office was so urgent as to justify the curtailment of the anti-tuberculosis work in the county. There can be little doubt as to the answer which will be given to this question. One does not need to be specially well-informed to learn that every medical man will be needed who can possibly be spared. At such a time as the present a much greater curtailment of anti-tuberculosis work must be viewed with such equanimity as other economies to be faced. The hope may, however, be expressed that accommodation for advanced cases will not be cut down. That would be not economy, but the collapse of the whole service.

### OVERCROWDING, POVERTY, AND SMALL OWNERSHIP.

In his annual report Dr. Sheppard, Medical Officer of Health for the New Forest, again draws attention to the difficulties experienced by Sanitary Authorities in cases of overcrowding. The policy of eviction under the Public Health Act is merely an evasion of the problem, and reminds us of the Elizabethan Poor Law, which dealt with its vagabonds by whipping them from one district to another. A solution of this difficulty is indeed much needed. Dr. Sheppard, however, from his long experience of this district with its examples of small ownerships and tenures, often historically interesting, demonstrates that these are also interesting from a sanitary point of view, on account of the superior economic status of the small owner. The problem of overcrowding is the problem of poverty, and no doubt the local encouragement of the smallholder would contribute to its solution.

### THE ADVANCED INSURANCE CONSUMPTIVE AND THE INFIRMARY.

Insured persons suffering from pulmonary tuberculosis in the London area are, as our readers know, sent for sanatorium or hospital treatment mainly to the Downs Sanatorium or to the Northern Hospital, Winchmore Hill. Those cases which are fairly advanced receive "hospital" treatment, and, as long as they make reasonable progress towards improvement, are allowed to remain several months at these institutions at the expense of the London Insurance Committee. Should they fail to improve, however, the rule is for them to be sent home again. The reason for this is twofold: on the one hand, the presence in a ward of a case which is going downhill has a depressing effect on the other patients who are hoping to get better; on the other hand, to allow these cases to remain until they die is to carry out a policy of segregation which hardly comes under the heading of treatment. From the public-health point of view the sending of advanced and not improving cases of consumption to their own homes is extremely bad policy, but the London Insurance Committee is concerned with treatment and not with questions of public health, which are directly the affair of the respective borough councils and the London County Council. The funds of the London Insurance Committee, it is justly considered, should not be used for an object which, however admirable, is one the expense for which should be borne by the ratepayers. A great many of the advanced cases above referred to eventually reach the Poor-Law infirmary, a large number remain at home and are a source of danger to other members of the family, and only a few find places in homes for the dying. The London County Council, it is true, is sending to sanatoria numbers of uninsured consumptives, and in respect of advanced cases is not unwilling to allow such cases to remain in hospitals until released by death. Would it not be a profitable policy, in view of what is known of the ways in which consumption is spread, for the London County Council to maintain in institutions those cases, even though insured, who have been discharged from hospital treatment as not likely to improve, and who have no proper accommodation at home and to whom there is no alternative but the workhouse infirmary?

### MERTHYR GENERAL HOSPITAL.

At the half-yearly meeting of the Governors of Merthyr General Hospital the workmen's representatives gave expression in forcible language to a grievance which seems to be keenly resented. For the half-year ending June 30, 1915, the excess of expenditure over ordinary receipts amounted to £522, and the report stated that the executive board at their meeting in May appointed two additional medical men upon the staff "in accordance with the concessions submitted and accepted by the workmen in February last." The report concluded with these words: "It is to be regretted that the workmen of the district cannot see their way clear to contribute a larger sum to meet the requirements of the institution." The workmen's representatives disputed the statement in the report that the desired concessions had been granted, and it was moved as an amendment that the words complained of be deleted. It appears that at Dowlais a medical man had, in fact, been appointed, but he was not the one the workmen wished to have, their delegates, it was alleged, having been out-voted by other representatives. It was stated that if the men's requirements had been carried out there would have been no contention as to contributions, but that if the Dowlais workmen did not get the doctor they wanted they would not subscribe. After much discussion the paragraph in question was taken out of the report, which was then adopted in its amended form. It is evident that the medical man whom the workmen desired to select enjoys considerable popularity among his patients, but it is unfortunate that the workmen should take the drastic step of withholding their contributions because their wishes in this respect were thwarted.

### CANADA'S SPLENDID CO-OPERATION: WAR DEMANDS FOR THE WOUNDED.

Last week we recorded that the Ontario Government are erecting a hospital at Orpington to contain 1,040 beds for acute cases among wounded men of the Canadian Contingent. We are indebted to the *Daily Telegraph*, which is doing yeoman service for the Empire by continuously dealing with the business side of the war and the needs and efforts of people of all classes throughout the Empire, for the following further interesting account of Canada's splendid record in providing equipment for the alleviation of suffering:—"The Canadian Red Cross Society, of which the Duke of Connaught is patron, is doing a magnificent work—thanks to the generous response to its appeals throughout Canada. The Society has borne the cost of the erection and equipment of the Duchess of Connaught Canadian Red Cross Hospital at Cliveden, Taplow, so that it may provide, with a smaller building which previously existed, accommodation for 1,000 patients; and for buildings and equipment the Society has devoted a sum of about £25,000. It has contributed £20,000 to the British Red Cross Society, about £2,000 to the St. John Ambulance Association, and a similar sum to the hospital train organised by Princess Christian. Only those who have been closely associated with the work of the women of the Dominion can appreciate their zeal and self-denial in providing comforts for Canadian soldiers at the Front. But in addition to their labours in this direction they have raised a sum of £50,000, which has been given to the War Office and Admiralty for Haslar Hospital ambulances. A sum of £20,000 has been devoted by the Dominion Government to the French hospital at Dinard, while 'La Presse' Hospital in Paris has been erected and maintained by the people of Quebec. With reference to the work of the Canadian Army Medical Corps, four general hospitals in France and England provide over 4,000 beds; stationary hospitals in France, in the Mediterranean, and in England have together nearly 8,000 beds. In addition, three casualty clearing stations in France and England provide for 600 patients, while two convalescent homes in the South of England have over 1,000 beds. In France there is also a Canadian Mobile Laboratory, as well as medical stores. In connection with Canadian efforts, reference should be made to the excellent work being done at the hospital at Beachborough Park, Shorncliffe, which is supported by Canadians." We hope that similar statistics will be forthcoming from Australia, India, New Zealand, South Africa, and each of the British Dominions beyond the seas, of which we should welcome the fullest information as soon as it is available. These are epoch-making days, and the present welding together of all races and Governments within the Empire is amongst the most momentous results for good now maturing.

### GOOD WORK IN NORFOLK.

With the object of assisting in the work of supplying British and Allied hospitals at home and abroad with general hospital requirements, a depôt has been established at Lowestoft in premises lent by Mr. B. Pareezer, proprietor of the Marina Theatre. There is a similar depôt at Norwich, in connection with which the Lowestoft branch will work. From Norwich, base hospitals in Alexandria, Malta, Serbia, and also many hospitals in France have been supplied by parcels sent from that city, and also in connection with the Norfolk and Suffolk Military Hospital the depôt has been able to supply a great deal of their needs. Subscriptions for the Lowestoft branch have been coming in well, but a special appeal for further funds is being made, and the manager has suggested that they could not do better than had been done in connection with the Norwich depôt—that is, secure a large number of vice-presidents with a subscription of as much as they pleased to give.

### MEDICAL MAN AS DAY LABOURER.

From medicine, as from other professions, there is an occasional lapse into some lower social rank as a result of misconduct or misfortune, but from Lancashire there comes a story of a doctor who adopted this descent deliberately and as a therapeutic measure. After some years of ill-health spent in various parts of the world the doctor in question, believing that physical work would benefit his health, took the position of an ordinary labourer in a Widnes foundry, and concealed his identity under an assumed name. On several occasions he was noticed to be particularly useful in rendering first aid in case of accidents, but his full professional status, so the story goes, was discovered when a broken limb which he had "set" was examined at the local hospital. Thereafter he practised in Widnes for some years and enjoyed a widespread popularity and respect, especially among the poorer classes of the community. Dr. Eustace—such was his name—graduated at Dublin in 1889, and his death occurred some few weeks ago.

### THIS WEEK'S DRUG MARKET.

Changes in the value of drugs have been somewhat numerous since the last report was written; and while the majority of these have been in an upward direction, the prices of a number of articles are not quite so firmly maintained. Among the synthetic drugs whatever alterations in prices there are, are in an upward direction, and so far as can be foreseen there is no probability of any reversal of this tendency for some time to come. Acetylsalicylic acid, barbitone, acetanilide, phenacetin, phenazone, and sulphonal, all tend upwards in price. Among other drugs which are again dearer can be numbered guaiacol carbonate, iron and quinine citrate, Curacao aloes, sandalwood oil, almond oil, thymol, benzoic acid, sodium benzoate, resorcin, lithia salts, and salol. Chamomiles are extremely scarce, and very high prices are quoted for the available supplies. A fair business has been done in quinine, and the value is firmly maintained. The easier tone in the quicksilver market has not affected the prices of mercurials. Holders of cod-liver oil show no inclination to reduce their quotations, and it looks as if buyers will have to become reconciled to excessively high prices. Citric acid, tartaric acid, essence of lemon, and menthol have an easier tendency in prices. There are indications that the scarcity of Epsom salts is likely to be relieved, and that prices will in consequence be somewhat lower.

